# A Prognostic Model of Triple-Negative Breast Cancer Based on miR-27b-3p and Node Status

**DOI:** 10.1371/journal.pone.0100664

**Published:** 2014-06-19

**Authors:** Songjie Shen, Qiang Sun, Zhiyong Liang, Xiaojiang Cui, Xinyu Ren, Huan Chen, Xiao Zhang, Yidong Zhou

**Affiliations:** 1 Department of Breast Surgery, Peking Union Medical College Hospital, Peking Union Medical College, Chinese Academy of Medical Sciences, Beijing, China; 2 Department of Pathology, Peking Union Medical College Hospital, Peking Union Medical College, Chinese Academy of Medical Sciences, Beijing, China; 3 Department of Surgery, Department of Obstetrics and Gynecology, Women’s Cancer Program, Samuel Oschin Comprehensive Cancer Institute, Cedars Sinai Medical Center, Los Angeles, California, United States of America; 4 Department of Microbiology, Zhejiang Institute of Microbiology, Hangzhou, Zhejiang, China; 5 Biostatistics and Bioinformatics Core, Samuel Oschin Comprehensive Cancer Institute, Cedars Sinai Medical Center, Los Angeles, California, United States of America; Georgetown University, United States of America

## Abstract

**Objective:**

Triple-negative breast cancer (TNBC) is an aggressive but heterogeneous subtype of breast cancer. This study aimed to identify and validate a prognostic signature for TNBC patients to improve prognostic capability and to guide individualized treatment.

**Methods:**

We retrospectively analyzed the prognostic performance of clinicopathological characteristics and miRNAs in a training set of 58 patients with invasive ductal TNBC diagnosed between 2002 and 2012. A prediction model was developed based on independent clinicopathological and miRNA covariates. The prognostic value of the model was further validated in a separate set of 41 TNBC patients diagnosed between 2007 and 2008.

**Results:**

Only lymph node status was marginally significantly associated with poor prognosis of TNBC (*P* = 0.054), whereas other clinicopathological factors, including age, tumor size, histological grade, lymphovascular invasion, P53 status, Ki-67 index, and type of surgery, were not. The expression levels of miR-27b-3p, miR-107, and miR-103a-3p were significantly elevated in the metastatic group compared with the disease-free group (*P* value: 0.008, 0.005, and 0.050, respectively). The Cox proportional hazards regression analysis revealed that lymph node status and miR-27b-3p were independent predictors of poor prognosis (*P* value: 0.012 and 0.027, respectively). A logistic regression model was developed based on these two independent covariates, and the prognostic value of the model was subsequently confirmed in a separate validation set. The two different risk groups, which were stratified according to the model, showed significant differences in the rates of distant metastasis and breast cancer-related death not only in the training set (*P* value: 0.001 and 0.040, respectively) but also in the validation set (*P* value: 0.013 and 0.012, respectively).

**Conclusion:**

This model based on miRNA and node status covariates may be used to stratify TNBC patients into different prognostic subgroups for potentially individualized therapy.

## Introduction

Triple-negative breast cancer (TNBC), which lacks expression of estrogen receptor (ER), progesterone receptor (PR), and HER-2, is characterized by poor prognosis and extreme heterogeneity [1], [2]. Some patients with TNBC experience early distant metastasis and particularly poor survival in the first 3–5 years following diagnosis. However, for others, mortality wanes such that at 10 years post diagnosis, these patients have a better survival than patients with luminal-type (ER+) tumors [3]–[5]. This suggests that patients with TNBC can be separated into two clinically distinct groups: those likely to experience an early metastasis and succumb to their disease in the first 3–5 years after diagnosis, and those expected to show excellent long-term survival [6].

Traditionally, a number of clinicopathological characteristics, such as patient age, tumor size, histological grade, hormone receptor status, HER-2 status, lymphovascular invasion and lymph node involvement, have been used to determine the prognosis of breast cancer patients [6]. However, with the exception of lymph node involvement, those traditional markers are of little value in predicting the prognosis of TNBC patients [7]–[9]. The lack of highly sensitive and specific prognostic markers is a major obstacle to predicting prognosis and planning individualized treatment in TNBC patients. Some multigene signatures, like the 70-gene MammaPrint signature, the PAM50 assay (Prosigna), and the 21-gene Oncotype Dx, have demonstrated their prognostic value in breast cancers. Despite the success of these signatures in hormone receptor-positive (HR+) breast cancer, the majority of TNBCs are highly proliferative and classified as having a poor prognosis signature [10]–[13]. Thus, these signatures failed to show notable value in discriminating prognosis within this subtype. Although some studies have identified multi-gene signatures that predict prognosis of TNBC [6], [14], [15], the reported signatures, composed of dozens of genes, are usually costly and based on frozen tissue, which diminishes their value for clinical application.

MicroRNAs (miRNAs) are a family of small non-coding RNA molecules that are considered to be master regulators of many important biological processes such as cancer cell growth, apoptosis, invasion, and metastasis [16]–[18]. miRNA expression signatures have been shown to be promising biomarkers for predicting outcomes in a wide array of human cancers [19]. Although many miRNAs have been identified as important predictors of breast cancer prognosis, to date, the prognostic value of miRNAs has rarely been investigated specifically in TNBC [20]–[24]. Distant metastasis is the major determinant of cancer mortality and the most reliable representative of prognosis. Therefore, we aimed to identify miRNAs that correlate with distant metastasis of TNBC and to establish a cost-effective and reliable prediction model. This model may be used to stratify TNBC patients into different prognostic subgroups for potentially individualized therapy.

## Materials and Methods

### Patients

A total of 99 TNBC patients were eligible and enrolled from our database between September 2002 and March 2012, including 58 for training and 41 for validation of the prediction model. All the patients underwent surgery and treatment at the breast department of the Peking Union Medical College Hospital (PUMCH). The validation set was restricted to patients who underwent mastectomy between 01/2007 and 12/2008, and the rest were included in the training set. The inclusion criteria were: (1) sample selection was limited to the most common histologically invasive ductal breast carcinoma (IDC); (2) status of ER, PR and HER-2 was available and negative; (3) patients had complete follow-up histories; (4) disease-free patients were followed up for at least 5 years; and (5) there was adequate tissue volume in the tissue bank for the study. Patients with local advanced or metastatic breast cancer at diagnosis, bilateral or inflammatory breast cancer, neoadjuvant treatment before surgery, or local recurrence before distant metastasis were excluded. The histological diagnosis and status of ER, PR, and HER-2 were re-tested centrally and confirmed by two pathologists independently according to the American Society of Clinical Oncology/College of American Pathologists guidelines [25], [26]. The following antibodies were used for immunohistochemistry (IHC): ER (M7047, clone 1D5), PR (M3569, clone 636), and HER-2 (A0485, polyclonal rabbit antibody), which were all purchased from Dako, Denmark. If Her-2 was 2+ by IHC, fluorescence in situ hybridization (FISH) analysis using a dual-color probe (PathVysion, Vysis Inc., USA) was required to confirm negativity. All the patients received adjuvant chemotherapy based on anthracyclines and/or taxanes. Radiation therapy was given to patients who had more than three positive axillary lymph nodes or received breast conserving surgery. No patients received endocrine or trastuzumab treatment. Radiographic imaging was conducted immediately after the diagnosis of breast cancer, then every 6 months during the first 2 years following surgery and every 12 months beyond 2 years after surgery.

The study was approved by the Institutional Review Board of the PUMCH. Written informed consent was given by participants for their clinical records and tissue samples to be used in this study.

### Tumor RNA Samples

Tissues were fixed in 10% buffered formalin and embedded in paraffin immediately after resection. For the 99 selected breast cancer patients, sections from the formalin-fixed paraffin-embedded (FFPE) primary tumor blocks were stained with hematoxylin and eosin (HE) stain to identify the IDC regions. Then, about a 0.1-cm^3^ tissue core was obtained from the IDC region of each FFPE block in order to minimize contamination from adjacent tissues. Total RNA was extracted from these tumor tissue cores for miRNA expression profiling.

### Real-Time Reverse Transcription PCR (RT-PCR) Detection of miRNAs

Total RNA was isolated from tumor tissue cores with the RecoverAll Total Nucleic Acid Isolation Kit (Ambion, USA) according to the protocol. The specific stem–looped RT-PCR primers for different miRNAs were designed according to a previous study [27] and are summarized in Table S1. The RT-PCR reaction was performed as previously reported [22]. PCR primers for amplification of mature human miRNAs are also summarized in Table S1.

Real-time PCR analysis was performed on an ABI 7500 instrument (ABI Inc., USA) with 20-µL reaction volumes containing 1 µL reverse transcription product, 10 µL 2X SYBR Green Mix (Invitrogen), 0.8 µL paired specific primers (10 µM), and 8.2 µL H_2_O. The reactions were incubated in 96-well plates at 95°C for 5 min, followed by 40 cycles of amplication (95°C for 15 s, 60°C for 31 s). They were then ramped from 60°C to 95°C to obtain the melting curve [22]. U6 snRNA was measured by the same method and used for normalization. The relative quantity (RQ) of each miRNA was calculated using the equation RQ = 2^−ΔΔCT^ [28], where CT is the threshold cycle to detect fluorescence.

### Statistical Considerations

Patients who developed distant metastasis as the first event within 5 years after removal of the primary tumors were considered to have poor prognosis, whereas patients who remained disease free for a minimum of 5 years were defined as having good prognosis. Time to distant metastasis (TTDM) was defined as the time from the breast cancer surgery to the earliest occurrence of distant metastasis. Time to death was defined as the time from the breast cancer surgery to breast cancer-specific death.

All immunohistochemical and molecular analyses were performed blinded to clinical data. Due to the non-normality of miRNA expression, the Mann–Whitney U test was performed for the analyses of miRNA expression between the metastatic group and the disease-free group. The cut-off values between high- and low-expression level of prognostic miRNAs were determined from the receiver operating characteristic (ROC) curve based on the highest Youden’s index (sensitivity+ specificity–1).

The Cox proportional hazards regression analyses were conducted to evaluate the association of clinicopathological factors and miRNAs to the distant metastasis-free (DMF) survival. To build an outcome prediction model using these independent predictor factors, we employed the logistic regression model. The Kaplan-Meier curves were produced for the DMF survival and overall survival (OS) of patients stratified by the prediction model. The *P* values of the survival differences in the Kaplan-Meier analyses were calculated by the log-rank test.

Statistical analyses were performed using the SPSS software (version 19.0: IBM SPSS, IL, USA), with a *P* value of less than 0.05 as the threshold of significance. All statistical tests were two-sided.

## Results

### Clinicopathological Characteristics and Association with TNBC Prognosis

Among the 58 primary TNBC patients in the training set, 31 developed distant metastasis with a median TTDM of 16 months (range: 2–59 months); and 27 remained disease-free with median follow-up of 68 months (range: 60–127 months). The median survival time from metastasis to death was only 7 months (range: 1–114 months). Survival results were last updated in August, 2013. The most common sites at initial presentation of distant metastases were lung (41.9%), bone (35.5%), liver (29.0%), and brain (22.6%). Nearly half (5/11, 45.5%) of bone metastases occurred concurrently with visceral metastases. Other locations were lymph nodes (6.5%) and skin (3.2%). There were 14 deaths following distant metastases, and no deaths happened in the DMF group. The clinicopathological characteristics of patients are listed in Table 1. The median age was 46.5 years (range: 25–79 years), and 32 (55.2%) patients were under age 50. All the patients were diagnosed as having triple-negative invasive ductal carcinomas. The tumor sizes of 26 (44.8%) patients were less than 2 cm, and in 22 (37.9%) patients were node negative. Most (89.7%) of the patients received modified radical mastectomy (MRM), and only 6 (10.3%) patients were treated by breast conserving surgery (BCS). The prognostic value of each clinicopathological factor related to DMF survival was analyzed using a Cox proportional hazards regression method. This part of the analysis is presented in Table 1. It shows that only lymph node status was marginally associated with DMF survival [hazard ratio (HR): 2.206, 95% confidence interval (CI): 0.985–4.942; *P* = 0.054], whereas other factors, including patient age at diagnosis, tumor size, histological grade, lymphovascular invasion, P53 status, Ki-67 index (14% as threshold [29]) and type of surgery, were not statistically significant predictors.

**Table 1:** Clinicopathological characteristics and association with distant metastasis of TNBC in the training set.

	No. of patients (n = 58)	No. of events (n = 31)	HR (95% CI)	*p-*value
Age at diagnosis (y)				0.875
≤50	32	17	1	
>50	26	14	0.945 (0.466–1.918)	
Tumor size (cm)				0.300
≤2	26	12	1	
>2	32	19	1.466 (0.711–3.022)	
Histological grade				0.275
G1	5	2	1	
G2	14	6	1.252 (0.252–6.210)	
G3	28	17	2.340 (0.538–10.166)	
Undefined	11	6	N/A	
LVI				0.907
No	54	29	1	
Yes	4	2	0.918 (0.219–3.851)	
P53				0.970
Negative	24	14	1	
Positive	26	14	1.014 (0.483–2.130)	
Undefined	8	3	N/A	
Ki-67 index*				0.349
<14%	6	2	1	
≥14%	32	18	2.013 (0.466–8.695)	
Undefined	20	11	N/A	
Lymph node status				0.054
Negative	22	8	1	
Positive	36	23	2.206 (0.985–4.942)	
Type of surgery				0.231
MRM	52	27	1	
BCS	6	4	1.901 (0.664–5.444)	

HR: hazard ratio; y: years; CI: confidence interval; LVI: lymphovascular invasion; MRM: modified radical mastectomy; BCS: breast conserving surgery; N/A: not applicable.

*Ki-67 index threshold of 14% was chosen according to the St. Gallen Consensus 2013 [29].

### miRNA Expression Profiles and Association with TNBC Survival

Next, we evaluated the prognostic value of miRNAs by expression profiling in the training set. First, total RNA samples were isolated from 58 FFPE primary breast cancer tissues. The expression profiles of five miRNAs (miR-21-5p, miR-27b-3p, miR-103a-3p, miR-107, and miR-210) were compared between 31 distant metastatic disease patients and 27 disease-free patients by RT-PCR. These five miRNAs were selected based on bioinformatics analysis of public miRNA microarray data, mining of public literature [20]–[24], as well as our preliminary results from testing the expression of these five miRNAs in a pilot study (unpublished data). These selected miRNAs have been implicated in a variety of cancers in previous studies, but none have been specifically investigated in TNBC. We utilized the Mann–Whitney U test to compare the expression level of each miRNA between the metastatic disease and the disease-free groups. The results are listed in Table 2. It shows that expression levels of miR-27b-3p and miR-107 were significantly elevated in the metastatic disease group compared with the disease-free patients (fold change: both 1.83; *P* value: 0.008 and 0.005, respectively). The expression level of miR-103a-3p was marginally elevated in the distant metastatic disease patients (fold change: 1.49; *P* = 0.050).

**Table 2:** Expression profile comparisons of the five selected miRNAs between 31 distant metastases patients and 27 disease-free patients in the training set by the Mann–Whitney U test.

miRNA Name	Good prognosis group		Poor prognosis group		ΔΔCT	Fold change	Up/Down	p-value
	ΔCT AVERAGE	ΔCT StDev	ΔCT AVERAGE	ΔCT StDev				
miR-21–5p	0.11	1.65	−0.33	0.93	−0.44	1.36	Up	0.591
miR-27b-3p	3.25	1.25	2.38	1.02	−0.87	1.83	Up	0.008
miR-103a-3p	4.81	1.29	4.23	1.04	−0.58	1.49	Up	0.050
miR-107	10.76	1.20	9.89	0.91	−0.87	1.83	Up	0.005
miR-210	5.59	1.65	5.21	1.32	−0.38	1.30	UP	0.538

CT: the threshold cycle to detect fluorescence; ΔCT = CT_miRNA_−CT_U6_, ΔΔCT = ΔCT_poor prognosis_−ΔCT_good prognosis_.

Patients who developed distant metastasis as first event within 5 years after removal of the primary tumor were considered to have poor prognosis, whereas patients who remained disease free for a minimum of 5 years were defined as having good prognosis. Fold change represented the miRNA expression level of poor prognosis group versus good prognosis group, and was calculated using the equation RQ = 2^−ΔΔCT^ [28].

Thus, miR-27b-3p and miR-107, as well as miR-103a-3p were included in the following analysis. The cut-off values of prognostic miRNAs were determined from the ROC curve to ensure the highest Youden’s index. An AUC (area under ROC curve) value of 0.5 indicates predictive performance that is no better than chance, whereas values greater than 0.5 indicate true predictive capacity. The AUCs of miR-27b-3p, miR-107, and miR-103a-3p were 0.705 (95%CI: 0.566–0.844; *P* = 0.008), 0.714 (95%CI: 0.581–0.847; *P* = 0.005), and 0.650 (95%CI: 0.506–0.794; *P* = 0.050), respectively (Figure S1). The cut-off value of miR-27b-3p was determined to be 3.553, with a sensitivity of 93.5% and specificity of 51.9% (Youden’s index 0.454). The cut-off value of miR-107 was 10.845, with a sensitivity of 90.3% and specificity of 48.1% (Youden’s index 0.385). The cut-off value of miR-103a-3p was 5.120, with the sensitivity, specificity, and Younden’s index being 83.9%, 48.1%, and 0.320, respectively. According to the cut-off value of each selected miRNA, patients were divided accordingly into a high-expression group and a low-expression group. The results of the Cox proportional hazards regression analyses of each miRNA related to DMF survival are listed in Table 3. Tumors with high miR-27b-3p expression had significantly increased risk of distant metastasis compared to low miR-27b-3p expression tumors (HR: 8.212, 95%CI: 1.953–34.529; *P* = 0.004). miR-107 and miR-103a-3p were also correlated to the risk of distant metastasis, with HR of 5.214 (95%CI: 1.580–17.207; *P* = 0.007) and 2.894 (95%CI: 1.108–7.557; *P* = 0.030), respectively.

**Table 3:** Univariate and multivariate Cox proportional hazards regression analysis to evaluate the independent prognostic value of clinicopathological parameters and miRNAs in relation to distant metastasis.

	No. of patients(n = 58)	No. of events(n = 31)	Univariate analysis		Multivariate analysis	
			HR (95% CI)	*p-*value	HR (95% CI)	*p-*value
LN status				0.054		0.012
Negative	22	8	1		1	
Positive	36	23	2.206 (0.985–4.942)		2.915 (1.262–6.736)	
miR-27b-3p				0.004		0.027
Low	16	2	1		1	
High	42	29	8.212 (1.953–34.529)		6.651 (1.239–35.691)	
miR-107				0.007		0.180
Low	16	3	1		1	
High	42	28	5.214 (1.580–17.207)		2.773 (0.624–12.323)	
miR-103a-3p				0.030		0.518
Low	18	5	1		1	
High	40	26	2.894 (1.108–7.557)		0.672 (0.202–2.242)	

HR: hazard ratio; LN: lymph node.

### Independent Prognostic Value of Clinical Parameters and miRNAs

According to the above univariate analyses, miR-27b-3p, miR-107 and miR-103a-3p, together with lymph node status as a clinical variable, were all related to poor prognosis of TNBC. We employed the multivariate Cox proportional hazards regression analysis to investigate their independence as prognostic factors of DMF survival in 58 TNBC patients (Table 3). The multivariate analysis indicated that lymph node status and miR-27b-3p were independent significant predictors of DMF survival, with the HR of 2.915 (95%CI: 1.262–6.736; *P* = 0.012) and 6.651 (95%CI: 1.239–35.691; *P* = 0.027), respectively. Each miR-107 and miR-103a-3p was associated with DMF survival, but neither was significant if adjusted by other prognostic factors (*P* value: 0.180 and 0.518, respectively), suggesting that these two miRNAs were not independent prognostic factors.

**Figure 1:**
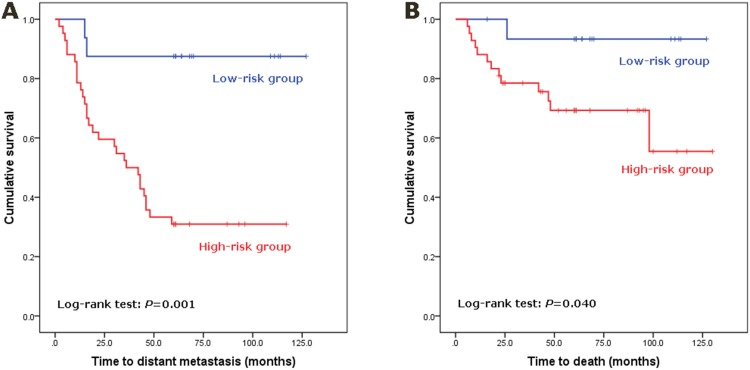
Kaplan-Meier analysis to evaluate the statistical power of the predictive model, based on miR-27b-3p and node status, in predicting the distant metastasis-free survival and overall survival of 58 TNBC patients in the training set. According to the prediction model, each patient was assigned to a low-risk or high-risk group. Significant differences in survival between groups were determined by log-rank analysis. (A) Application of the model to predict the distant metastasis outcomes of TNBC patients. (B) Application of the model to predict the overall survival of TNBC patients.

We then applied the logistic regression to lymph node status and miR-27b-3p expression level to build a prediction model. Both lymph node status and miR-27b-3p were significant in the logistic regression analysis, with an odds ratio of 4.773 (95%CI: 1.264–18.019; *P* = 0.021) and 21.181 (95%CI: 3.747–119.734; *P* = 0.001), respectively. The coefficients (±standard error) of node status and miR-27b-3p were 1.563 (±0.678) and 3.053 (±0.884), respectively, with the constant of −3.117 (±0.964). Thus, we built a logistic regression model for prognosis prediction in TNBC as follows: S = 1.563×V_LN+3.053×V_miR-27b-3p−3.117, where S represents the risk score for each patient, V_LN for lymph node status (0 for lymph node negative and 1 for positive), and V_miR-27b-3p for the expression levels of miR-27b-3p (0 for low expression level and 1 for high expression level) in each patient. According to the definition of the estimated probability (p) = exp(S)/(1+exp(S)) in the logistic regression, four probability values can be generated: 0.042, 0.175, 0.484, and 0.817. Thus, we defined 0.042 and 0.175 as representing low risk of distant metastasis, whereas 0.484 and 0.817 represent a high risk of distant metastasis. Forty-two patients were predicted to be of high risk by the model, with 16 patients of low risk. There were 29 cases of distant metastases and 13 deaths in the high-risk group, while 2 cases of metastases and 1 death were observed in the low-risk group. The estimated sensitivity, specificity, positive predictive value (PPV), and negative predictive value (NPV) of distant metastases were 93.5%, 51.9%, 69.0%, and 87.5%, respectively. The Kaplan-Meier survival analysis indicated that this model was significantly predictive of DMF survival for the 58 TNBC patients (*P* = 0.001; Figure 1A). The prediction model also showed a significantly correlation with overall survival (*P* = 0.040; Figure 1B). The estimated sensitivity, specificity, PPV, and NPV of death were 92.9%, 34.1%, 31.0%, and 93.8%, respectively.

### Model Validation with an Independent TNBC Cohort

**Figure 2:**
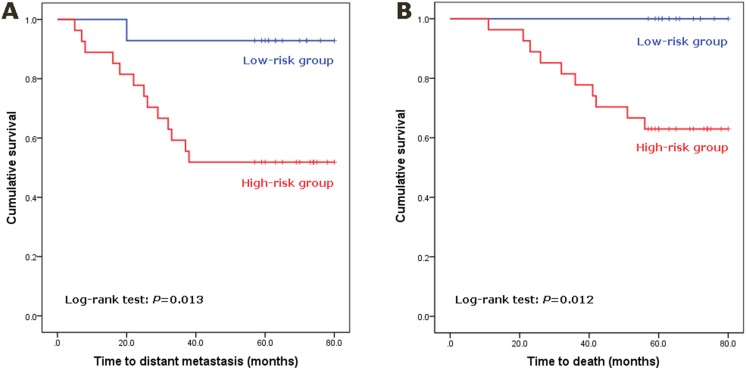
Prognostic performance of the prediction model in a validation cohort of 41 TNBC patients. A risk score was assigned to each patient as calculated by the prediction model. Based on the risk score, the patients were stratified into either the low-risk group or the high-risk group. Kaplan-Meier analysis and log-rank tests were used to determine the significant differences in survival between groups. (A) Differences in distant metastasis-free survival between low-risk and high-risk groups. (B) Differences in overall survival between low-risk and high-risk groups.

The prediction model was further validated in an independent series of 41 TNBC patients who received mastectomy between January 2007 and December 2008. The median follow-up was 61 months (range: 11–80 months), resulting in 14 (34.1%) cases of distant metastases and 10 (24.4%) deaths. The clinicopathological characteristics of the validation cohort are listed in Table 4. The Cox proportional hazards regression analysis showed that none of the clinicopathological factors were statistically associated with DMF survival, while the lymph node status had a moderate effect (HR: 2.606, 95% CI: 0.903–7.522; *P* = 0.077). The expression level of miR-27b-3p was determined in the 41 validation samples by RT-PCR and also turned out to be significantly correlated with the risk of distant metastasis (HR: 8.487, 95% CI: 1.109–64.982; *P* = 0.039). A risk score was then calculated for each of the 41 TNBC patients by applying the combination of miR-27b-3p expression levels and lymph node status to the prediction model. Based on the risk score, 27 patients were classified into the high-risk group, and 14 patients into the low-risk group. There were 13 cases of distant metastases and 10 deaths in the high-risk group, while only one case of metastasis and no death occurred in the low-risk group. The estimated sensitivity, specificity, PPV, and NPV of distant metastases were 92.9%, 48.1%, 48.1%, and 92.9%, respectively. Kaplan-Meier analysis indicated that the two stratified patient groups had significantly different DMF survival rates (*P* = 0.013; Figure 2A). These two patient groups were also significantly different in overall survival (*P* = 0.012; Figure 2B). The estimated sensitivity, specificity, PPV, and NPV of death are 100%, 45.2%, 37.0%, and 100%, respectively.

**Table 4:** Univariate analysis of clinicopathological characteristics and miR-27b-3p expression with distant metastasis of TNBC in a validation set.

	No. of patients (n = 41)	No. of events (n = 14)	HR (95% CI)	*p-*value
Age at diagnosis (y)				0.908
≤50	18	6	1	
>50	23	8	1.065 (0.369–3.069)	
Tumor size (cm)				0.708
≤2	22	7	1	
>2	19	7	1.222 (0.428–3.485)	
Histological grade				0.882
G1	5	2	1	
G2	13	4	0.675 (0.124–3.688)	
G3	20	7	0.867 (0.180–4.180)	
Undefined	3	1	N/A	
LVI				0.330
No	38	12	1	
Yes	3	2	2.112 (0.470–9.500)	
P53				0.135
Negative	25	11	1	
Positive	16	3	0.377 (0.105–1.354)	
Ki-67 index				0.559
<14%	9	4	1	
≥14%	32	10	0.708 (0.222–2.258)	
Lymph node status				0.077
Negative	25	6	1	
Positive	16	8	2.606 (0.903–7.522)	
miR-27b-3p				0.039
Low	14	1	1	
High	27	13	8.487 (1.109–64.982)	

HR: hazard ratio; y: years; CI: confidence interval; LVI: lymphovascular invasion; N/A: not applicable.

## Discussion

TNBC is an aggressive but heterogeneous subtype of breast cancer. It is imperative to stratify TNBC using new tools to improve the prognostication and help understand the biological basis of TNBC progression. Here we compared the clinicopathological factors and miRNA expression profiles between an early-distant-metastasis patient cohort and a disease-free survival patient cohort. Lymph node status and miR-27b-3p expression were found to be independent predictors for distant metastasis of TNBC. By a logistic regression of these two covariates, we defined a prognostic model, which significantly stratified distant metastasis-free survival and overall survival of TNBC in both the training set and an independent validation set.

TNBC accounts for about 15–20% of all breast cancers and is characterized by a special pattern of recurrence [3], [4], [30], [31]. Compared with other subtypes of breast cancer, TNBC has a much higher risk of recurrence, especially distant metastasis, which peaks at approximately 3 years after the initial diagnosis and declines so rapidly thereafter that it seldom occurs beyond 5 years [3], [4], [30]. TNBC has a higher incidence of visceral metastases and cerebral metastases [31], which is also true in our cohort with 41.9% lung, 29.0% liver and 22.6% brain metastases. TNBC patients with distant metastases have an extremely poor prognosis, with a median survival time from recurrence to death of less than 1 year (7 months in this study), which is significantly shorter than that of women with other types of breast cancer [4]. Despite TNBC patients having a high risk of early metastases, it seems that women without recurrence in the first 5 years are unlikely to die of breast cancer [1], [4], [31], [32]. Frustratingly, there are few, if any, clinicopathological variables showing prognostic capacity to stratify the two clinically distinct groups. Previous reports suggest that factors such as tumor size, tumor grade, extent of vascular invasion, P53 status, and patient age show little relationship to patient outcome in the context of TNBC [7], [33]. Besides the above mentioned factors, we also found types of surgery and Ki-67 levels to be of no predictive value in TNBC. Indeed, the only clinicopathological variable that was prognostic in TNBC appeared to be lymph nodal status in the present study (*P* = 0.054), which is consistent with other reports [7]–[9]. However, in patients with TNBC, once there is evidence of lymph node metastasis, the prognosis may not be affected by the number of positive lymph nodes [9]. Thus, node status was a weak prognostic factor, which was also only marginally associated with DMF survival (*P* = 0.077) in the validation set.

Furthermore, node status only represents anatomical stage differences between two groups, but the two clinically distinct TNBC subgroups must be intrinsically or genetically different [32]. A few multi-gene signatures were reported to predict prognosis of TNBC [6], [14], [15]; however, gene profiling is not cost-effective or convenient for clinical application. miRNAs are considered to be master regulators of many important biological processes [16]–[18]. It was shown that formalin fixation and paraffin embedding did not change the stability of miRNA, and the expression profiles of miRNA were in good correlation between matched fresh frozen and FFPE samples [34], [35]. miRNA can be detected in FFPE samples, which makes it more convenient to apply in the clinic. Many miRNAs have been identified as important prognostic predictors of breast cancer [20]–[24]. However, to our knowledge, the prognostic value of miRNAs has rarely been investigated specifically in TNBC. Some studies have established a few miRNA signatures that are associated with prognosis of TNBC, but those studies only tested miRNA signatures in the patient cohort in which the signatures were generated [36]–[38]. Herein, we found that miR-27b-3p, miR-107, and miR-103a-3p are prognostic for DMF survival of TNBC, and miR-27b-3p is an independent predictor among other miRNAs and clinical covariates. In human breast cancer, miR-103 and miR-107 were reported to target Dicer and promote cell migration and invasion by induction of epithelial-to-mesenchymal transition (EMT) [21]. miR-27b is encoded in an imprinted region of chromosome 9 in humans and belongs to the family of miR-27 [39]. miR-27b directly targets peroxisome proliferator-activated receptors γ (PPARγ), and functions as an oncogene in breast cancer in a cell-type dependent style [39], [40]. High expression of miR-27b was reported to be associated with poor prognosis of breast cancer, and silencing of miR-27b reduced tumor growth and metastases [20], [39]. Subgroup analysis of 37 patients in one of the studies also hinted that miR-27b was prognostic exclusively in ER-negative tumors, especially in TNBC, but it was not further validated [20].

We hypothesize that the miR-27b-3p determines the genetic ability of tumors to invade and metastasize, irrespective of the stage, while lymph node status reflects the mobilization of tumor cells and presence of metastasis from the primary site. Thus, the combination of the two factors may generate a robust model of distinguishing between TNBC with good and poor outcomes. By logistic regression, the model based on miR-27b-3p expression and node status was developed to divide TNBC patients into high risk and low risk groups. The two stratified groups showed significantly different prognosis of distant metastasis and breast cancer-related death not only in the training set (*P* value: 0.001 and 0.040, respectively) but also in the validation set (*P* value: 0.013 and 0.012, respectively).

Taken together, these findings demonstrate the capacity of our prediction model to stratify TNBC patients into different prognostic subgroups for potentially individualized therapy. The most important value of the model lies in the identification of patients in the high risk category who are likely to develop distant metastatic disease despite standard local and systemic therapy; these patients should be referred to clinical trials that include novel therapeutic strategies. Our study also indicates the need for further investigation into the role of miR-27b in TNBC progression and metastasis.

## Supporting Information

Figure S1 Receiver operating characteristic (ROC) curve of miR-27b-3p, miR-107, and miR-103a-3p to predict the distant metastasis of patients in the training set (n = 58).
(TIF)

Table S1 PCR primers for amplification of the muture human miRNAs.
(DOC)
