# Supplementary figures and images for: A Prognostic Model of Triple-Negative Breast Cancer Based on miR-27b-3p and Node Status
Source: PLoS One. 2014 Jun 19;9(6):e100664. doi: 10.1371/journal.pone.0100664 (PMC4063964; doi:10.1371/journal.pone.0100664)

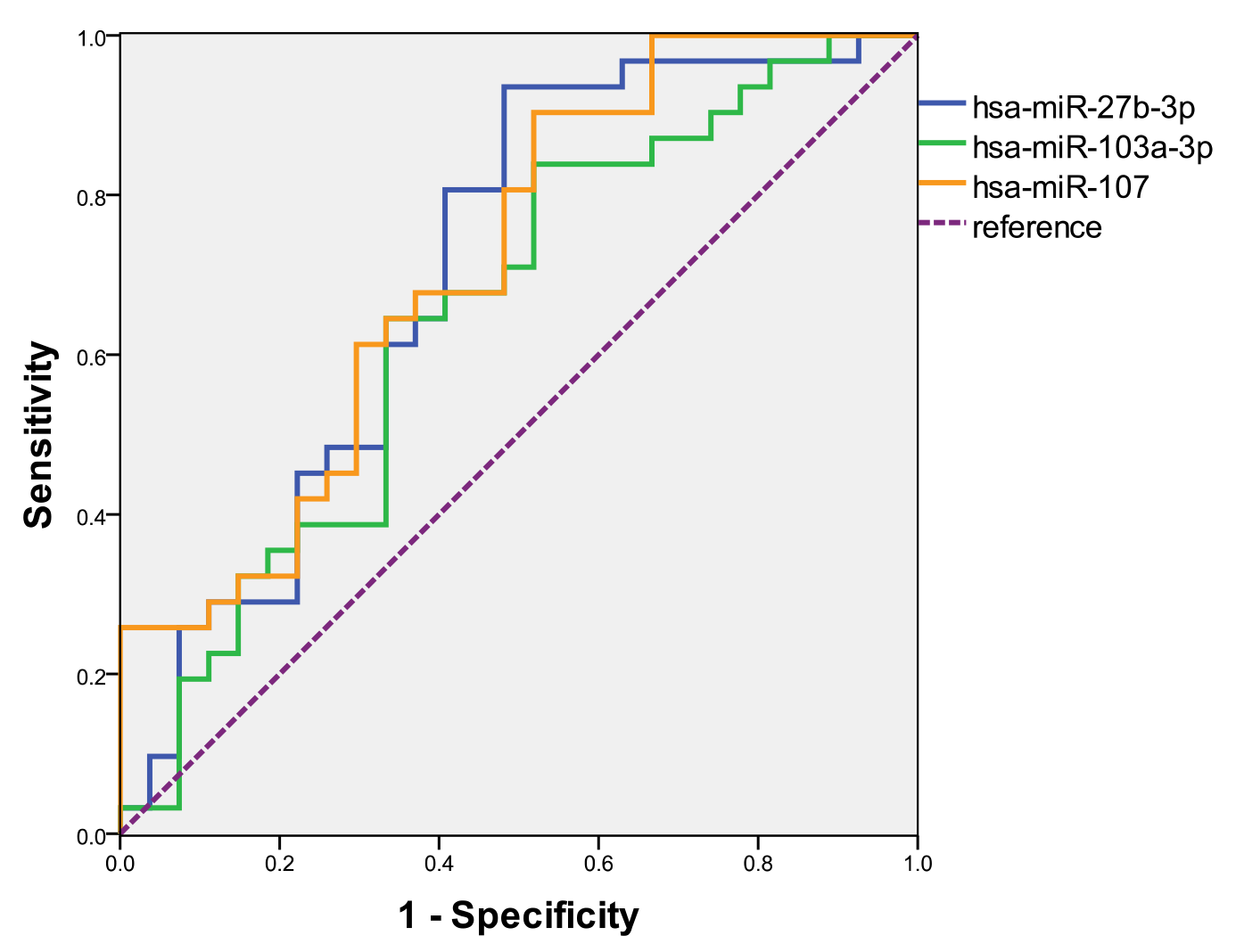

Supplement: Figure S1 — Receiver operating characteristic (ROC) curve of miR-27b-3p, miR-107, and miR-103a-3p to predict the distant metastasis of patients in the training set (n = 58). (TIF) [file pone.0100664.s001.tif]
