# Supplementary material for: A Prognostic Model of Triple-Negative Breast Cancer Based on miR-27b-3p and Node Status
Source: PLoS One. 2014 Jun 19;9(6):e100664. doi: 10.1371/journal.pone.0100664 (PMC4063964; doi:10.1371/journal.pone.0100664)
Supplement: Table S1 — PCR primers for amplification of the muture human miRNAs. (DOC) [file pone.0100664.s002.doc]

**Table S1.** PCR primers for amplification of the muture human miRNAs.

| miRNA name | Primer sequence (5’>3’) |
| --- | --- |
| hsa-miR-21-RT | GTCGTATCCAGTGCAGGGTCCGAGGTATTCGCACTGGATACGACTCAACA |
| hsa-miR-21-F | CGGGCGTGTAGCTTATCAGACT |
| hsa-miR-103-RT | GTCGTATCCAGTGCAGGGTCCGAGGTATTCGCACTGGATACGACTCATAG |
| hsa-miR-103-F | CCAGCGTGAGCAGCATTGTA |
| has-miR-107-RT | GTCGTATCCAGTGCAGGGTCCGAGGTATTCGCACTGGATACGACTGATAG |
| has-miR-107-F | CGGGCGTGAGCAGCATTGTA |
| hsa-miR-27b-3p-RT | GTCGTATCCAGTGCAGGGTCCGAGGTATTCGCACTGGATACGACGCAGAA |
| hsa-miR-27b-3p-F | GGCGTGTTCACAGTGGCTAAG |
| hsa-miR-210-RT | GTCGTATCCAGTGCAGGGTCCGAGGTATTCGCACTGGATACGACTCAGCC |
| hsa-miR-210-F | AGAGCGTGCTGTGCGTGTG |
| Consensus primer R | CAGTGCAGGGTCCGAGGTATT |

(RT):reverse transcript primer, (F): forward primer, (R): reverse primer.
